# Characteristics of Microbial Community and Function With the Succession of Mangroves

**DOI:** 10.3389/fmicb.2021.764974

**Published:** 2021-12-07

**Authors:** Zhimao Mai, Mai Ye, Youshao Wang, Swee Yeok Foong, Lin Wang, Fulin Sun, Hao Cheng

**Affiliations:** ^1^State Key Laboratory of Tropical Oceanography, Key Laboratory of Tropical Marine Bio-Resources and Ecology, South China Sea Institute of Oceanology, Chinese Academy of Sciences, Guangzhou, China; ^2^Southern Marine Science and Engineering Guangdong Laboratory (Guangzhou), Guangzhou, China; ^3^Guangdong Provincial Academy of Environmental Science, Guangzhou, China; ^4^School of Biological Sciences, Universiti Sains Malaysia, Penang, Malaysia; ^5^Daya Bay Marine Biology Research Station, Chinese Academy of Sciences, Shenzhen, China

**Keywords:** mangrove succession, microbial community and function, carbon metabolism, *Rhizophora apiculata*, Merbok river estuary

## Abstract

In this study, 16S high-throughput and metagenomic sequencing analyses were employed to explore the changes in microbial community and function with the succession of mangroves (*Sonneratia alba*, *Rhizophora apiculata*, and *Bruguiera parviflora*) along the Merbok river estuary in Malaysia. The sediments of the three mangroves harbored their own unique dominant microbial taxa, whereas *R. apiculata* exhibited the highest microbial diversity. In general, Gammaproteobacteria, Actinobacteria, Alphaproteobacteria, Deltaproteobacteria, and Anaerolineae were the dominant microbial classes, but their abundances varied significantly among the three mangroves. Principal coordinates and redundancy analyses revealed that the specificity of the microbial community was highly correlated with mangrove populations and environmental factors. The results further showed that *R. apiculata* exhibited the highest carbon-related metabolism, coinciding with the highest organic carbon and microbial diversity. In addition, specific microbial taxa, such as Desulfobacterales and Rhizobiales, contributed the highest functional activities related to carbon metabolism, prokaryote carbon fixation, and methane metabolism. The present results provide a comprehensive understanding of the adaptations and functions of microbes in relation to environmental transition and mangrove succession in intertidal regions. High microbial diversity and carbon metabolism in *R. apiculata* might in turn facilitate and maintain the formation of climax mangroves in the middle region of the Merbok river estuary.

## Introduction

Mangroves represent one of the most productive ecosystems in tropical and subtropical estuaries and shorelines. They possess biological resources and play important roles in carbon fixation, erosion mitigation, and water purification (Giri et al., 2011; Brander et al., 2012). They often occur in marine-terrestrial ecotones with obvious geographical and hydrological heterogeneities, leading to interesting sequential species zonation along continuous gradients. The adaptation and succession of mangroves in intertidal regions are speculated to be closely related to microbes in the sediments. Unfortunately, the potential roles of microbes and their functions in mangrove ecosystems are still poorly understood, although changes in vegetation during mangrove succession and how mangrove plants adapt to intertidal environmental adversities have been well studied (Wang et al., 2019; Cheng et al., 2020).

In recent years, high-throughput sequencing has offered a comprehensive perspective of microbes (Andreote et al., 2012; Alzubaidy et al., 2016; Lin et al., 2019). Benthic microbial community is highly correlated with soil properties (the depth of soil layer, pH, salinity, and nutrient availability) (Abed et al., 2015; Zhou et al., 2017; Tong et al., 2019) and aboveground plants (Bardgett and van der Putten, 2014). Previous findings also showed that microbial diversity and function might vary significantly among different mangrove habitats because of environmental transition and mangrove succession (Bai et al., 2013). Mangrove coverage also regulates the structure and composition of microbial community by altering redox conditions and organic carbon levels in the sediments (Holguin et al., 2001).

Moreover, microbes in sediments also play important biogeochemical roles (e.g., C, N, and S cycles), which can facilitate mangrove survival in intertidal regions (Reis et al., 2017). Owing to the withering and retention of mangrove branches and leaves, mangrove sediments contain a large amount of organic carbon, and most carbon turnover in mangrove ecosystems is carried out by sediment microbes (Alongi, 1988). Benthic microbes may also promote the efficiency of biogeochemical cycles in the sediments, such as C, N, and S cycles (Lin et al., 2019). In addition, anaerobic metabolism can further facilitate the production and consumption of methane and nitrous oxide (Giani et al., 1996; Reis et al., 2017), which can contribute to the emission of greenhouse gases from mangrove wetlands (Rosentreter et al., 2018). However, the responses of microbes to environmental transition and mangrove succession have not been well demonstrated. It is essential to further identify microbial taxa and their metabolic potential in mangrove ecosystems.

Thus, the present study aimed to (i) identify the changes in microbial community and diversity among different mangrove habitats; (ii) explore microbial functions and metabolic potentials in mangrove ecosystems; and (iii) explore the potential correlations among environmental factors, mangrove populations, and benthic microbial communities and functions. The purpose of this study was to evaluate the hypothesis that microbial community and function would respond positively to environmental transitions and might contribute to mangrove survival and succession in intertidal regions. Therefore, surface sediments from three mangrove fields (*Sonneratia alba*, *Rhizophora apiculata*, and *Bruguiera parviflora*) were examined using 16S high-throughput and metagenomic sequencing analyses. The implications of this study should be useful for guiding future research on the roles of the microbial community and their functions in mangrove succession.

## Materials and Methods

### Study Area and Sample Collection

**FIGURE 1:**
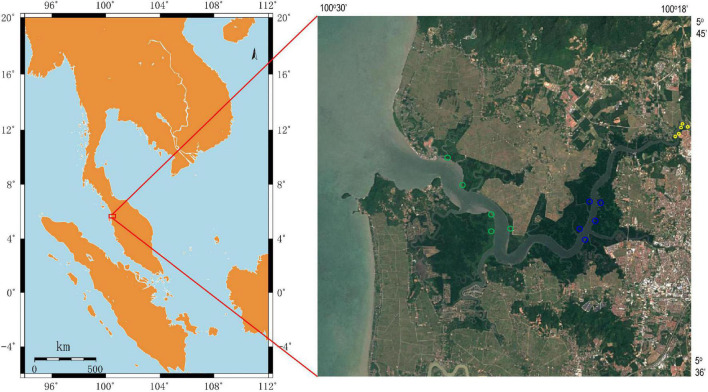
Location of sampling sites in Merbok river estuary, Malaysia. Sediment samples were collected from *Sonneratia alba* (yellow circle), *Rhizophora apiculata* (blue circle) and *Bruguiera parviflora* (green circle).

Sediment samples were collected on November 25, 2019, in a mangrove reserve located in the Merbok river estuary, Malaysia (Figure 1). In the upper estuary (with lower salinity), the habitats were mainly occupied by *S. alba* and sporadically mixed with *Nypa fruticans*, whereas the lower estuary had groves of *B. parviflora* and sporadic *Avicennia* genera. In the middle region of the Merbok river estuary, the dominant species was *R. apiculata*. According to tidal transition and mangrove succession, surface sediments were collected from three mangrove populations (*S. alba*, *R. apiculata*, and *B. parviflora*), and five parallel samples were collected from each mangrove population. The samples were placed in liquid nitrogen and frozen for nucleic acid extraction. The chemical parameters of the sediments were determined and described as follows.

### Determination of the Chemical Parameters of Mangrove Sediments

The sediment properties included salinity, pH, total organic carbon (TOC), total phosphorus (TP), and total nitrogen (TN). Salinity and pH were measured during sampling in the field. The samples were dried naturally and sieved (2 mm). TOC, TP, and TN in the sediments were then analyzed following standard measurements (Huang et al., 2017).

### Illumina Sequencing of 16S rRNA Gene and Data Analysis

Sediment samples (1 g) were weighed, and total DNA was extracted using an E.Z.N.A.^®^ Soil DNA Kit (Omega, Norcross, GA, United States). In this study, the V3 and V4 regions of the 16S rRNA gene were sequenced using 338F (5′-ACTCCTACGGGAGGCAGCAG-3′) and 806R (5′-GGACTACHVGGGTWTCTAAT-3′) primers. PCR was performed in a 25 μl reaction containing the following: 5 × *FastPfu* buffer (4 μl), 2.5 mM of dNTPs (2 μl), 5 U/μl of *FastPfu* polymerase (0.5 μl), 5.0 μM of primers (1.0 μl each), and template DNA (10 ng). PCR was performed using the following conditions: pre-denaturation at 95°C for 3 min; 30 cycles of denaturation at 95°C for 30 s, annealing at 55°C for 30 s, and extension at 72°C for 45 s; and a final extension at 72°C for 10 min. After amplification, the PCR products were purified and analyzed by paired-end sequencing (2 × 250) using the Illumina MiSeq platform (Illumina, San Diego, CA, United States) according to standard protocols. All sequences were deposited in the National Center for Biotechnology Information (NCBI) Sequence Read Archive (SRA) Database under accession number PRJNA756333.

For the paired-end reads obtained by MiSeq sequencing, samples were distinguished according to the barcode information and then spliced according to overlap sequences using FLASH (version 1.2.11). After quality control analysis, normalization of clean data was carried out for operational taxonomic unit (OTU) clustering analysis and species taxonomy analysis. CD-HIT tool was used to define tags with sequence similarities >97% as OTU clusters. QIIME software (version 1.9.1) was used to analyze the alpha diversities of the sequences, based on the Shannon index and ACE index. A representative sequence (showing the highest default abundance) in each OTU was selected for species classification using RDP classifier software (version 2.11) with the default threshold 0.8. Two-sided Welch’s *t*-test was used to analyze the statistical significances of microbial community structure in different samples. Beta diversity was calculated using unweighted UniFrac, and principal coordinates analysis (PCoA) was performed with R software (version 3.3.1). Redundancy analysis (RDA) was performed using the R software (version 3.3.1) vegan package, and statistical significances of RDA were judged by performing the PERMUTEST analysis of variance.

### Metagenomic Sequencing Analysis

Qualified DNA samples were diluted in fragmentation buffer and randomly disrupted using a Covaris M220 ultrasonicator (Covaris, Inc., Woburn, MA, United States). The DNA fragments obtained after disruption were used for library construction. The qualified library was sequenced using the Illumina HiSeq 2500 high-throughput sequencing platform with 2 × 150 paired-ends. This platform was also used for data configuration and image analysis with HiSeq Control software. Metagenomic data have been deposited in the NCBI SRA Database (PRJNA766709).

Raw sequence data were trimmed with FASTP^1^, and low-quality reads, lengths of < 50 base pairs, N-rich reads, and adapter reads were removed. Sequences with different sequencing depths were assembled using Megahit software^2^, and contigs were obtained using the succinct de Bruijn graph method. MetaGene (Noguchi et al., 2006) was used to predict open reading frames (ORFs) in contigs, and a statistical table of gene predictions was obtained for each sample. The predicted gene sequences of all samples were clustered by CD-HIT^3^, and the longest gene from each cluster was selected as the representative sequence to construct a non-redundant gene set. With the use of SOAPaligner (Li et al., 2009), the high-quality reads of each sample were compared with the non-redundant gene set (95% identity) to determine the abundance information for genes in corresponding samples. BLASTP (version 2.2.28) was used to compare the non-redundant gene set with the NR database (*e*-value: 1e^–5^), and species-annotation results were obtained based on the corresponding taxonomy information of the NR database. The analysis of Kyoto Encyclopedia of Genes and Genomes (KEGG) was used for functional annotation according to the BLAST results. Community contributions to functions were determined using the NR database annotation.

## Results

### Sediment Physicochemical Parameters

The differences in physicochemical properties among the three mangrove populations are shown in Supplementary Figure 1. Overall, the highest TOC and TN levels were detected in the sediments of *R. apiculata* among the three mangroves studied (*p* < 0.05). Higher TP and pH, but lower salinity, were observed in the sediments of *S. alba* than *R. apiculata* and *B. parviflora*.

### 16S rRNA Gene Illumina MiSeq Sequencing

**FIGURE 2:**
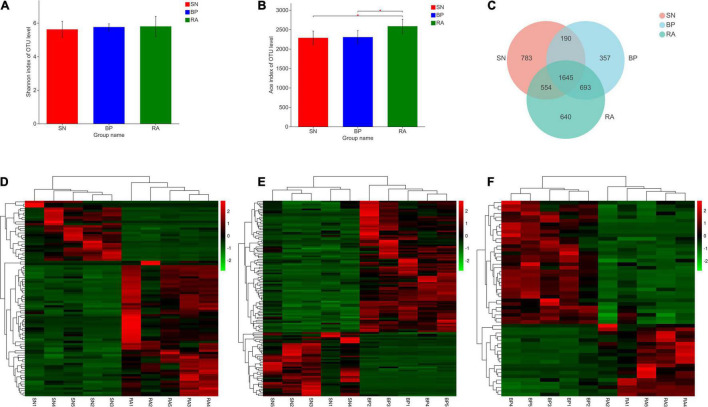
Diversity and significant difference analysis of operational taxonomic units (OTUs). **(A)** Shannon index of OTU level. **(B)** ACE index of OTU level. **(C)** Venn diagram analysis of OTU numbers between samples. **(D)** Significant difference analysis of OTUs between RA and SN. **(E)** Significant difference analysis of OTUs between BP and SN. **(F)** Significant difference analysis of OTUs between RA and BP. SN, *Sonneratia alba*; RA, *Rhizophora apiculata*; BP, *Bruguiera parviflora*.

Based on Illumina sequencing, 893,587 sequences were obtained from 15 samples. In total, 4,842 OTUs were observed at a 97% similarity level (Supplementary Table 1). In terms of microbial diversity, *R. apiculata* exhibited the highest OTUs number and bacterial richness index among the three mangrove populations (*p* < 0.05; Figure 2B). Higher Shannon index values were also represented for *R. apiculata*, although the differences were not significant. The Venn diagram shown in Figure 2C revealed a total of 3,532 OTUs in the sediments associated with *R. apiculata*, whereas 640 specific OTUs were observed. In addition, 1,645 common OTUs were observed among the three mangroves. As shown in Figures 2D–F, the types and relative abundances of dominant OTUs for *R. apiculata* and *B. parviflora* increased significantly compared with those for *S. alba*. Significant differences were also found in dominant OTUs between *R. apiculata* and *B. parviflora*.

### Bacterial Community Composition in Mangrove Sediments

**FIGURE 3:**
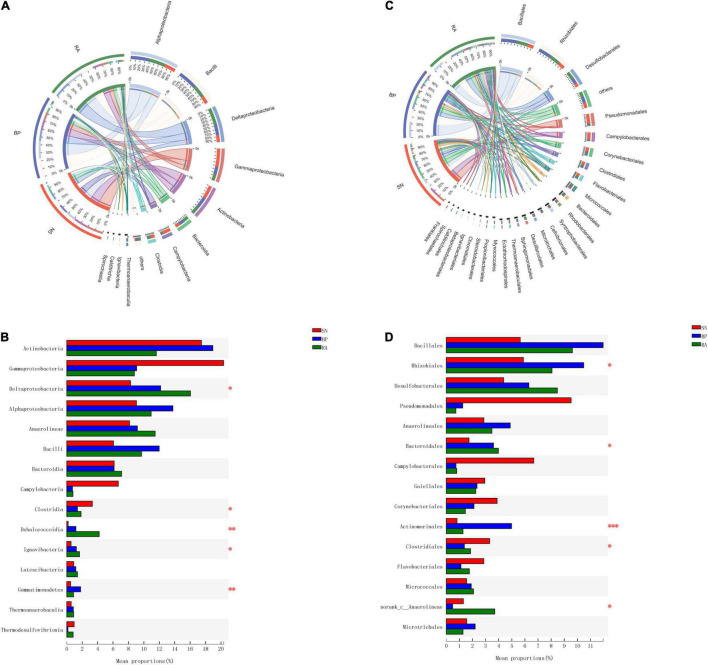
The components of bacterial taxa at class level **(A)** and order level **(B)**. **(C)** Significant difference analysis of dominant taxa at class level. **(D)** Significant difference analysis of dominant taxa at order level. SN, *Sonneratia alba*; RA, *Rhizophora apiculata*; BP, *Bruguiera parviflora*.

The abundances of bacterial taxa corresponding to the sediments are shown in Figure 3. Dominant bacteria (>5% at class level, Figure 3A) in mangrove sediments included Actinobacteria, Gammaproteobacteria, Deltaproteobacteria, Alphaproteobacteria, Anaerolineae, Bacilli, and Bacteroidia. However, the abundances of these dominant bacteria varied significantly among the three mangrove populations (Figure 3B). The abundances of Alphaproteobacteria, Deltaproteobacteria, and Bacilli were found to be higher for *R. apiculata* and *B. parviflora*, while the highest abundance of Gammaproteobacteria was observed in *S. alba* among the three mangroves.

The dominant bacterial taxa at the order level (Figure 3C) were Bacillales, Rhizobiales, Desulfobacterales, Pseudomonadales, Anaerolineales, and Bacteroidales. The highest abundances of Desulfobacterales were observed in the sediments associated with *R. apiculata*, followed by *B. parviflora* and *S. alba* (Figure 3D). Relative higher abundances of Bacillales and Rhizobiales were also detected in *R. apiculata* and *B. parviflora* than *S. alba*. In contrast, the highest abundance of Pseudomonadales was observed in *S. alba* among the three mangroves.

### Principal Coordinates Analysis and Redundancy Analysis of Microbial Community

**FIGURE 4:**
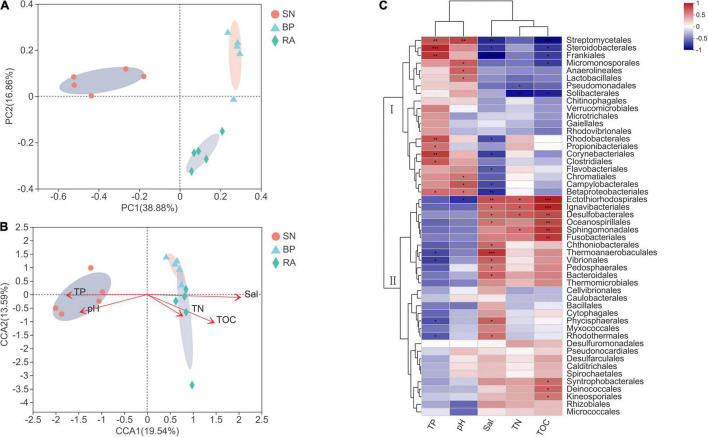
**(A)** Principal coordinates analysis (PCoA) of bacterial community structure. **(B)** Canonical correlation analysis of environmental factors and bacterial community. **(C)** Correlation analysis of dominant bacterial taxa and environmental factors. SN, *Sonneratia alba*; RA, *Rhizophora apiculata*; BP, *Bruguiera parviflora*.

The results from PCoA (Figure 4A) showed that microbial communities from the same mangrove population clustered together well. RDA was performed to explore the relationships between environmental parameters and microbial community (Figure 4B). The microbial communities with *R. apiculata* and *B. parviflora* were highly correlated with TOC, TN, and salinity, whereas bacteria with *S. alba* were highly affected by TP and pH. The results (Figure 4C) showed that the dominant microbial taxa were grouped into two clusters. Cluster I, including Chromatiales, Corynebacteriales, Rhodobacterales, and Anaerolineales, showed positive correlations with TP or pH (*p* < 0.05). Cluster II, including Desulfobacterales, Rhizobiales, and Micrococcales, exhibited positive trends for correlations with TOC, TN, and salinity. More detailed information of the dominant bacterial taxa at family level is shown in Supplementary Figure 2.

### Metagenomic Analysis of Microbial Community Function in Different Mangrove Sediments

**FIGURE 5:**
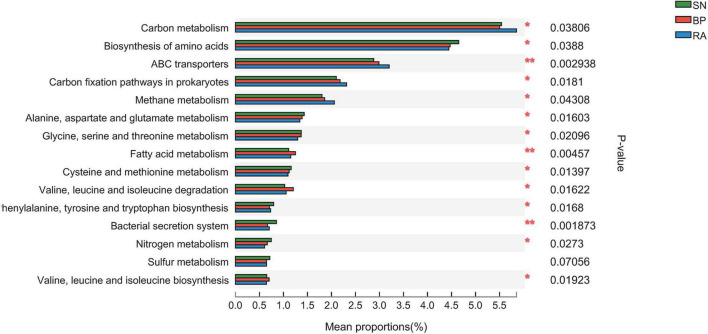
Significant difference analysis of metabolic function based on Kyoto Encyclopedia of Genes and Genomes (KEGG) function annotation. SN, *Sonneratia alba*; RA, *Rhizophora apiculata*; BP, *Bruguiera parviflora*.

Metagenome analysis generated a massive amount of sequence information, ranging from 90,898,652 to 139,622,818 reads among samples. In total, 14,664,335 non-redundant genes were detected in the metagenomes, and 9,005 KEGG Orthogroups (KOs) were identified. The results from Supplementary Figure 3 show a high correlation coefficient between the microbial community and functional diversity, with values of 0.84 and 0.87 for α diversity and β diversity, respectively. The data further illustrated the differences in key metabolic pathways (e.g., carbon-related metabolism) among the three mangroves. The sediments associated with *R. apiculata* exhibited the highest carbon metabolism, ABC transporters, prokaryote carbon fixation, and methane metabolism among the three mangroves (Figure 5 and Supplementary Figure 4).

### Contribution of Microbial Community to Kyoto Encyclopedia of Genes and Genomes Function

**FIGURE 6:**
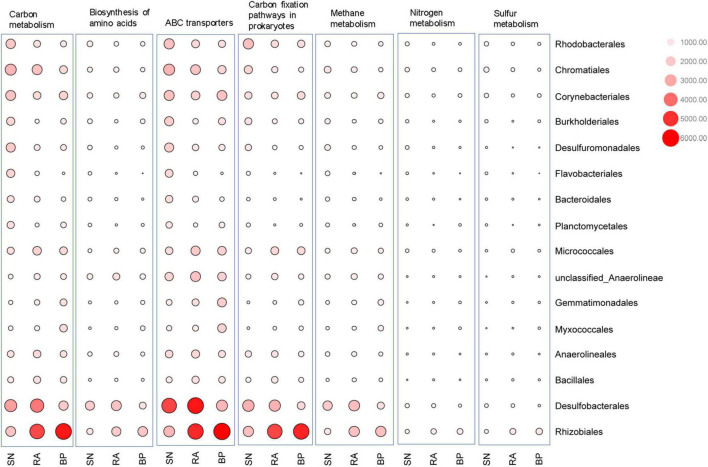
Contribution analysis of dominant microbial taxa to the function at order level. SN, *Sonneratia alba*; RA, *Rhizophora apiculata*; BP, *Bruguiera parviflora*.

As shown in Figure 6 and Supplementary Figure 5, Desulfobacterales (e.g., Desulfobacteraceae) and Rhizobiales (e.g., Xanthobacteraceae) were two major contributors to metabolic functions. The contribution of microbial taxa to the function varied significantly among the different mangrove species. Desulfobacterales consistently had the highest contributions to carbon-related metabolism (e.g., carbon metabolisms, prokaryote carbon fixation, methane metabolism, and ABC transporters) in the sediments associated with *R. apiculata*. When compared with *S. alba*, Rhizobiales also exhibited higher contributions to carbon-related metabolism in sediments of *R. apiculata* and *B. parviflora*. In addition to Desulfobacterales and Rhizobiales, the highest contributions of Micrococcales and Bacillales to metabolic functions were also observed in sediments associated with *R. apiculata*. In contrast, the lowest contributions of Corynebacteriales, Burkholderiales, and Planctomycetales to the aforementioned metabolic pathways were observed in the sediments associated with *R. apiculata*.

## Discussion

### Microbial Diversity Was Highly Affected by Tidal Transitions and Mangrove Succession

Significant differences were found in the microbial community among the three mangrove populations along the Merbok river estuary, and specific microbial communities were formed in each mangrove population (Figures 2, 3). Our previous data also indicated that microbial diversity was highly influenced by the structure of mangrove populations (Wu et al., 2016). Root exudates and secondary metabolites, which also could serve as carbon sources and antimicrobial substances, vary significantly among mangrove species (Gao et al., 2003; Koh et al., 2013). Changes in root exudates might not only affect benthic microbial density but also strongly affect the structure and function of microbes (Berendsen et al., 2012; Zhuang et al., 2020). Moreover, the present data showed that sediments associated with *R. apiculata* exhibited the highest microbial diversity. *R. apiculata* is a dominant mangrove species in South Asia and often develops into a stable and fully successional mangrove community. In this study, *R. apiculata* occupied the majority of ecological niches in the central Merbok river estuary, whereas the genera of *Avicennia*, *Bruguiera*, *Sonneratia*, and *Nypa* occupied a smaller habitat in the lower and/or upper estuary. Higher microbial diversity in sediments associated with *R. apiculata* suggested that mangrove succession could enrich benthic microbial community.

Coinciding with high microbial diversity, the sediments associated with *R. apiculata* also exhibited higher TOC than those with *B. parviflora* and *S. alba*, indicating a higher capacity for plant productivity, carbon fixation, and burial. As an important carbon source for microbes, previous investigators also claimed that the accumulation of organic matter in sediments could promote bacterial diversity (Sjöling et al., 2005; Chen et al., 2016). Moreover, the highest TN was also observed in sediments associated with *R. apiculata*. The present results were consistent with previous documents (Zhu et al., 2018), in which the shift in bacterial community structure was partly driven by the increased TOC and total organic nitrogen with the succession of mangrove. Nevertheless, other environmental parameters (salinity, pH, and TP) had significant effects on the microbial community (Figure 4B). Salinity and pH have already been reported to have large effects on the microbial communities of mangroves (Ikenaga et al., 2010; Chambers et al., 2016). Moderate salinity was also positively correlated with bacterial abundances and closely linked to community composition and diversity (Morrissey et al., 2014; Crespo-Medina et al., 2016; Tong et al., 2019).

### High Carbon-Related Metabolic Potential in Sediments Associated With *Rhizophora apiculata*

In this study, microbial diversity was significantly higher in sediments associated with *R. apiculata*, coinciding with the highest TOC. Enhanced microbial diversity would promote the transformation and utilization of organic carbon (Holguin et al., 2001; Sjöling et al., 2005; Berendsen et al., 2012). Thus, it is not surprising that the sediments associated with *R. apiculata* exhibited a higher carbon metabolic potential (Figure 5). Previous data also showed that bacterial diversity and metabolic potential (especially carbon metabolism) appeared to be enhanced during mangrove succession (Zhu et al., 2018).

The findings of this study further showed that sediments associated with *R. apiculata* also exhibited the highest abundances of genes involved in carbon-related pathways (Supplementary Figure 4). It is well known that several prokaryotes can assimilate CO_2_ into organic carbon (Lynn et al., 2017), although the functions of prokaryotes in carbon fixation have not been fully reported in mangrove ecosystems. In this study, Desulfobacterales and Rhizobiales exhibited high contributions in CO_2_ fixation. The enrichment of these bacteria could result in a higher capacity for prokaryotic carbon fixation, which plays an essential role in carbon storage.

It should be noted that the sediments of *R. apiculata* also exhibited the strongest potential for methane metabolism (Figure 5). In this study, the high organic carbon contents in *R. apiculata* sediments might promote the growth of methanogens, contributing to the potential production of CH_4_. Moreover, CH_4_ production and emission are aggravated under anaerobic conditions (Fey et al., 2004; Kutzbach et al., 2004). In addition, ABC transporters were also important indicators of microbial functions that reflected the positive activity of carbon and nutrient transformation. In this study, Desulfobacterales and Rhizobiales were the main contributors to ABC transporters. Higher abundances of ABC transporters in sediments associated with *R. apiculata* indicated that this mangrove had higher carbon and nutrient transportation activities than other mangroves (Wood et al., 2001).

Overall, this study suggested that climax mangroves (e.g., *R. apiculata*) exhibited a faster turnover rate of organic matter between plants and microbes owing to high carbon utilization and transportation. Microbial carbon fixation contributed to carbon sequestration, whereas the degraded small organic molecules could be conducive to the growth and succession of mangroves. The positive feedback of microbial community and function might in turn contribute to the formation of climax mangrove populations with high productivity (Chen et al., 2016). The inherent correlations among carbon metabolism, environmental transition, and mangrove succession need to be further studied.

### Specific Microbial Taxa and Functional Potential in Maintaining Mangrove Survival and Succession

Although mangrove sediments are rich in organic matter, they are generally nutrient-deficient. Nutrient limitations were also widely reported in mangrove forests (Feller et al., 2003; Reef et al., 2010; Wang and Gu, 2013; Cheng et al., 2020). Even worse, anaerobic conditions might further aggravate the enrichment of anaerobic microbes and reductive phytotoxins (e.g., CH_4_, H_2_S, and sulfides). It is worth exploring how mangroves maintain survival and succession in such a terrible habitat in intertidal regions. The present results could partly explain this issue from the perspectives of microbial community and function.

Desulfobacterales, a type of sulfate-reducing bacteria, consistently exhibited the highest contributions to metabolic functions in sediments associated with *R. apiculata* (Figure 6 and Supplementary Figure 5). In this study, Desulfobacterales (e.g., Desulfobacteraceae) was one of the main microbial taxa responsible for the differences among mangroves, whereas *R. apiculata* also exhibited the highest abundances of these bacteria (Figure 3D and Supplementary Figure 2A). Significantly positive correlations among Desulfobacterales, TOC, and TN were also observed (Figure 4C and Supplementary Figure 2B). Previous investigators also claimed an important role for Desulfobacterales in C, N, and S cycles (Zhu et al., 2018). Increased Desulfobacterales might be beneficial for *R. apiculata* by alleviating the toxicity of sulfides under anaerobic conditions. The high abundances and strong metabolic potential of Desulfobacterales could also accelerate carbon and nutrient transformation and utilization (Lyimo et al., 2002; Meyer and Kuever, 2007), facilitating mangrove survival and succession in intertidal regions.

The important potential of Rhizobiales in C, N, and S metabolisms was also revealed (Figure 6). Rhizobiales was a well-studied plant symbiont that widely occurred in the rhizosphere of mangrove plants (Gomes et al., 2010). This taxon played a beneficial role for the host by providing various nutrients, phytohormones, and precursors of essential metabolites (Delmotte et al., 2009; Verginer et al., 2010; Garrido-Oter et al., 2018). The findings of this study also revealed that Rhizobiales (e.g., Xanthobacteraceae) was metabolically versatile, especially in terms of carbon-related metabolism (Figure 6 and Supplementary Figure 5). In this study, relative higher abundances of Rhizobiales (e.g., Xanthobacteraceae) were also detected in the sediments of *R. apiculata* and *B. parviflora* than those of *S. alba* (Figures 2A, 3D). Similarly, positive correlation trends among Rhizobiales, TOC, and TN were also observed in this study (Figure 4C).

In addition to Desulfobacterales and Rhizobiales, Micrococcales might also partly contribute to the higher metabolic function in sediments associated with *R. apiculata*. Although little attention has been paid to this taxon, the present data (Figures 3D, 4C, 6) indicated that Micrococcales might also be important for metabolic functions, which are involved in carbon and nutrient metabolisms. Nevertheless, *Bacillus*, which was often considered a bionematicide, could promote the growth of plants by protecting roots from pathogens (Mendoza et al., 2008; Mendis et al., 2018). Bacillus was also a dominant bacterial component in this study (Figure 3D); however, relatively low metabolic potentials of this taxon were observed (Figure 6). Multi-omics analyses, such as metagenomics, metatranscriptomics, and metaproteomics, focused on the functions of microbes in mangrove ecosystems should be further conducted.

## Conclusion

The present findings provided a broader understanding of the relationships among microbes, environmental transition, and mangrove succession from the perspective of microbial community and function. Benthic microbial community was highly correlated with environmental factors and aboveground mangrove species, whereas the highest microbial diversity and metabolic potential (carbon metabolism, prokaryote carbon fixation, methane metabolism, and ABC transporters) were observed in sediments associated with *R. apiculata*. Specific microbial taxa (e.g., Desulfobacterales and Rhizobiales) involved in C, N, and S cycles might facilitate mangrove survival and succession in intertidal regions. The present data indicated that mangrove succession could enrich microbial diversity and carbon metabolism. More detailed multi-omics researches focused on the roles of microbes in mangrove succession should be further conducted.

## Data Availability Statement

The original contributions presented in the study are included in the article/Supplementary Material, further inquiries can be directed to the corresponding authors.

## Author Contributions

ZM carried out manuscript writing and revisions. FS and HC designed the research, writing, review, and editing; YW carried out writing – review. SF contributed to the sample collection and data analysis. LW carried out data analysis. All authors contributed to the article and approved the submitted version.

## Conflict of Interest

The authors declare that the research was conducted in the absence of any commercial or financial relationships that could be construed as a potential conflict of interest.

## Publisher’s Note

All claims expressed in this article are solely those of the authors and do not necessarily represent those of their affiliated organizations, or those of the publisher, the editors and the reviewers. Any product that may be evaluated in this article, or claim that may be made by its manufacturer, is not guaranteed or endorsed by the publisher.

## Footnotes

1
https://github.com/OpenGene/fastp

2
https://github.com/voutcn/megahit

3
http://www.bioinformatics.org/cd-hit
