# Supplementary material for: Characteristics of Microbial Community and Function With the Succession of Mangroves
Source: Front Microbiol. 2021 Dec 7;12:764974. doi: 10.3389/fmicb.2021.764974 (PMC8689078; doi:10.3389/fmicb.2021.764974)

Table S1 Sequence information and diversity index among different mangrove sediment. (SN, *S. alba*; RA, *R. apiculata*; BP, *B. parviflora*)

| Sample | Number | OTU number | Shannon | ACE | Chao |
| --- | --- | --- | --- | --- | --- |
| SN1 | 51815 | 1510 | 4.893315 | 2028.149 | 2082.548 |
| SN2 | 56676 | 1680 | 5.593268 | 2230.525 | 2331.427 |
| SN3 | 63165 | 1703 | 5.586512 | 2340.398 | 2420.867 |
| SN4 | 62674 | 1811 | 5.81905 | 2333.929 | 2346.924 |
| SN5 | 64330 | 1859 | 6.217083 | 2498.18 | 2537.326 |
| BP1 | 58993 | 1604 | 5.668817 | 2241.075 | 2299.519 |
| BP2 | 57264 | 1870 | 6.076595 | 2421.976 | 2377.691 |
| BP3 | 48997 | 1597 | 5.560236 | 2157.58 | 2153.012 |
| BP4 | 58901 | 1601 | 5.709313 | 2169.245 | 2166.216 |
| BP5 | 67099 | 1819 | 5.779439 | 2538.569 | 2520.99 |
| RA1 | 71879 | 1715 | 5.756607 | 2429.029 | 2412.594 |
| RA2 | 62712 | 1918 | 6.191945 | 2587.857 | 2652.115 |
| RA3 | 67864 | 2103 | 6.239404 | 2876.07 | 2981.51 |
| RA4 | 48333 | 1944 | 6.034267 | 2581.377 | 2574.217 |
| RA5 | 52885 | 1662 | 4.775743 | 2449.665 | 2424.438 |

Figure S1 Differences in physicochemical properties of sediments between mangrove populations. (SN, *S. alba*; RA, *R. apiculata*; BP, *B. parviflora*)


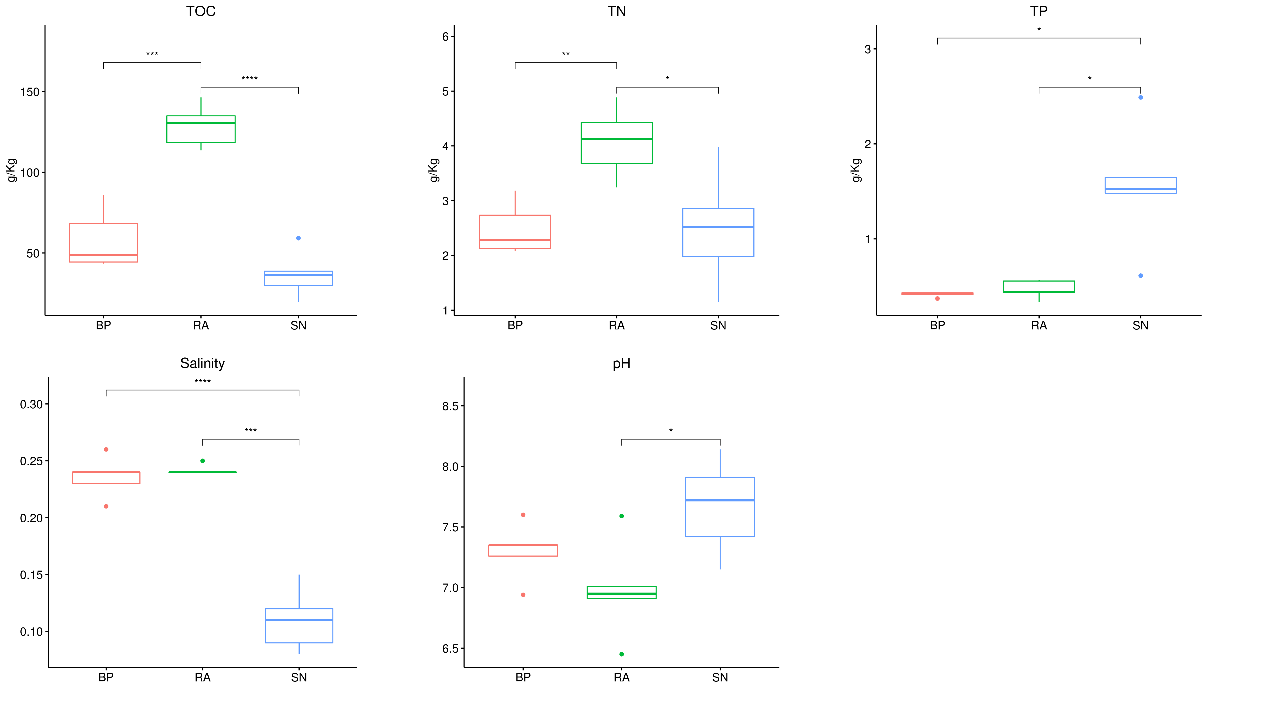


Figure S2 The taxa abundance of microbe corresponding to mangrove sediments in family level (A); Correlation analysis of dominant family taxa and environmental factors. (SN, *S. alba*; RA, *R. apiculata*; BP, *B. parviflora*).


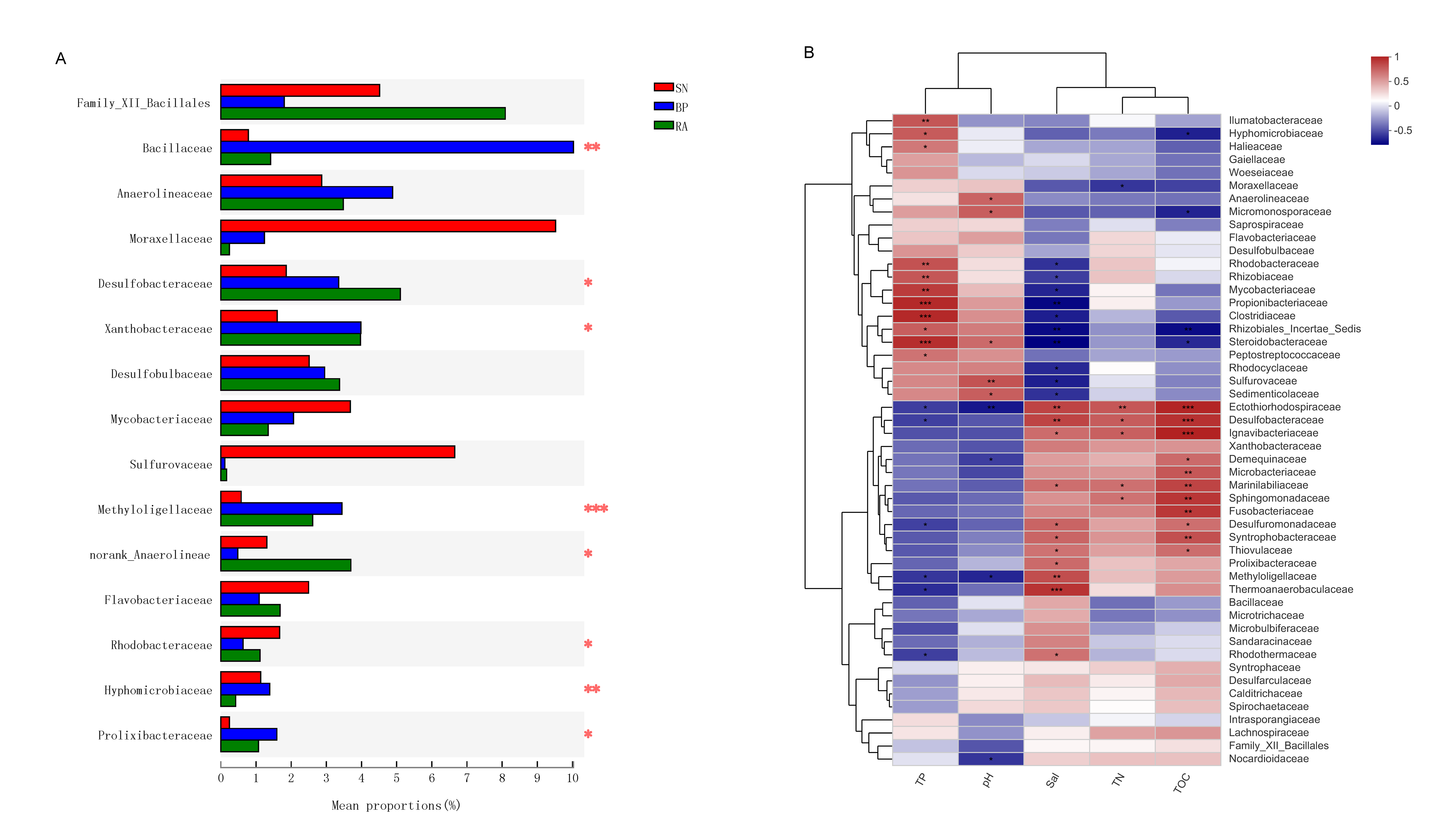


Figure S3 Analysis of correlation coefficient between microbial community and function in α diversity (A) and β diversity (B). (SN, *S. alba*; RA, *R. apiculata*; BP, *B. parviflora*)


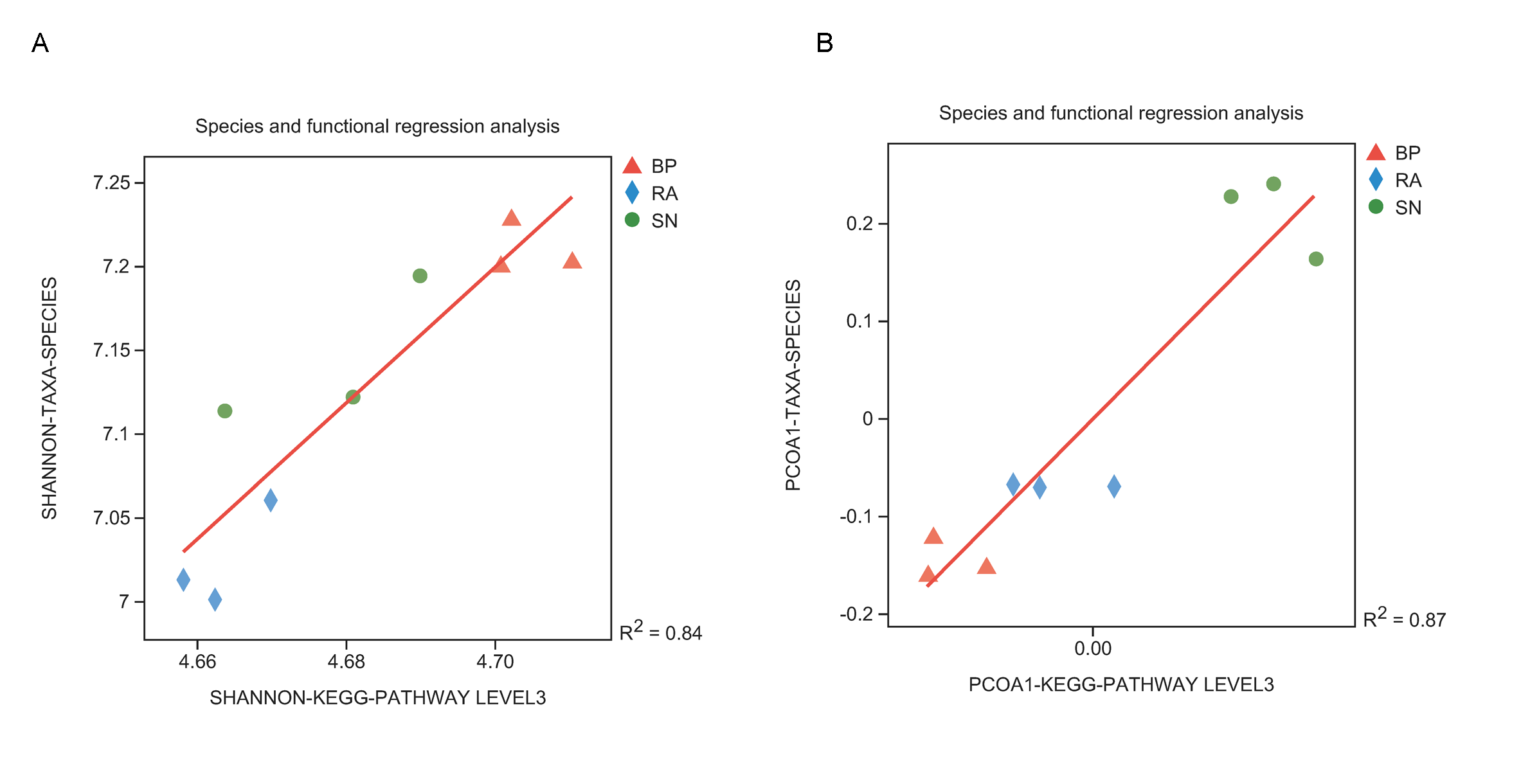


Figure S4 Comparison of carbon-related metabolism among three mangrove species. (SN, *S. alba*; RA, *R. apiculata*; BP, *B. parviflora*).


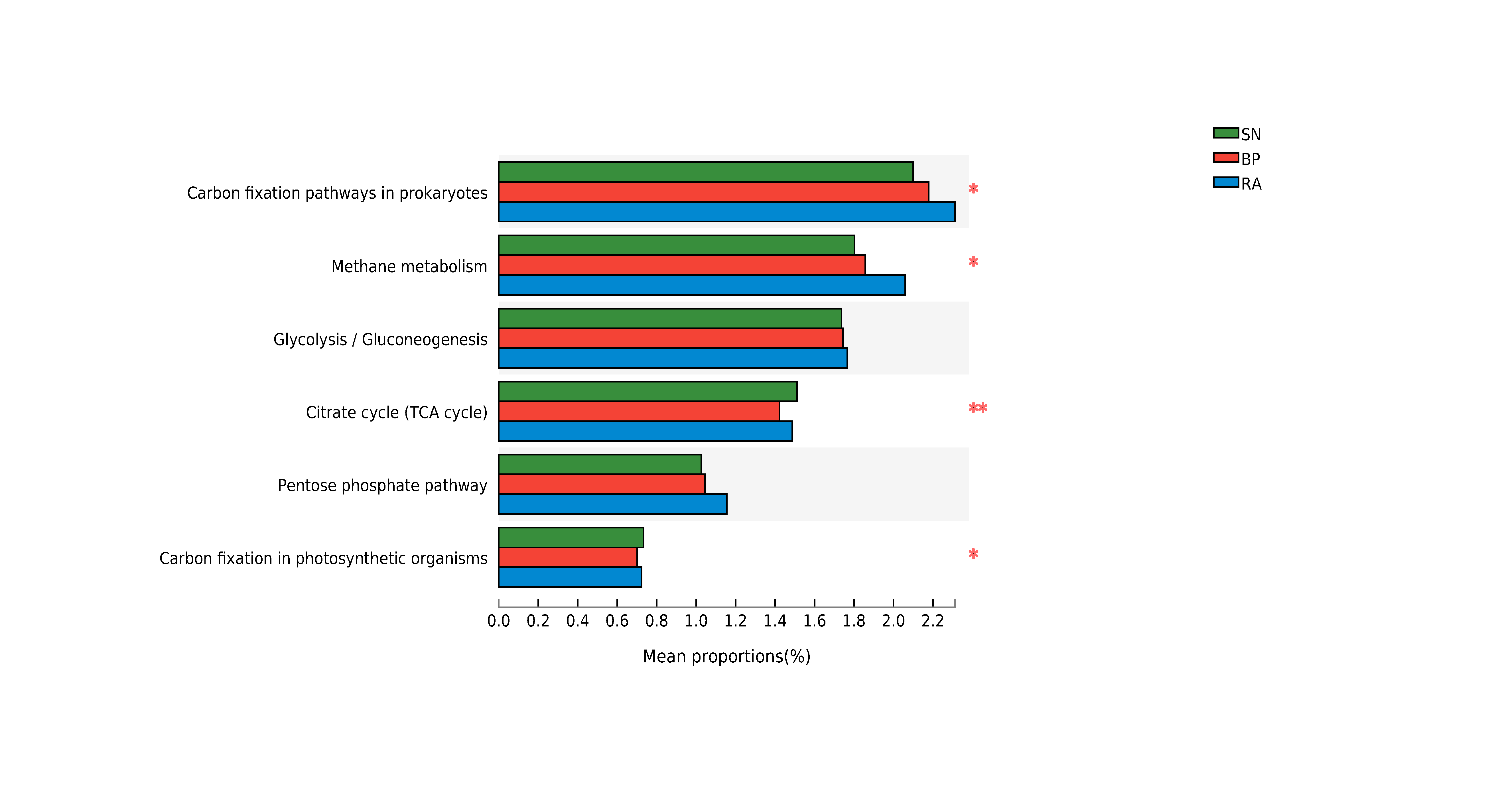


Figure S5 Contribution analysis of dominant microbial taxa to the function at family level. (SN, *S. alba*; RA, *R. apiculata*; BP, *B. parviflora*)


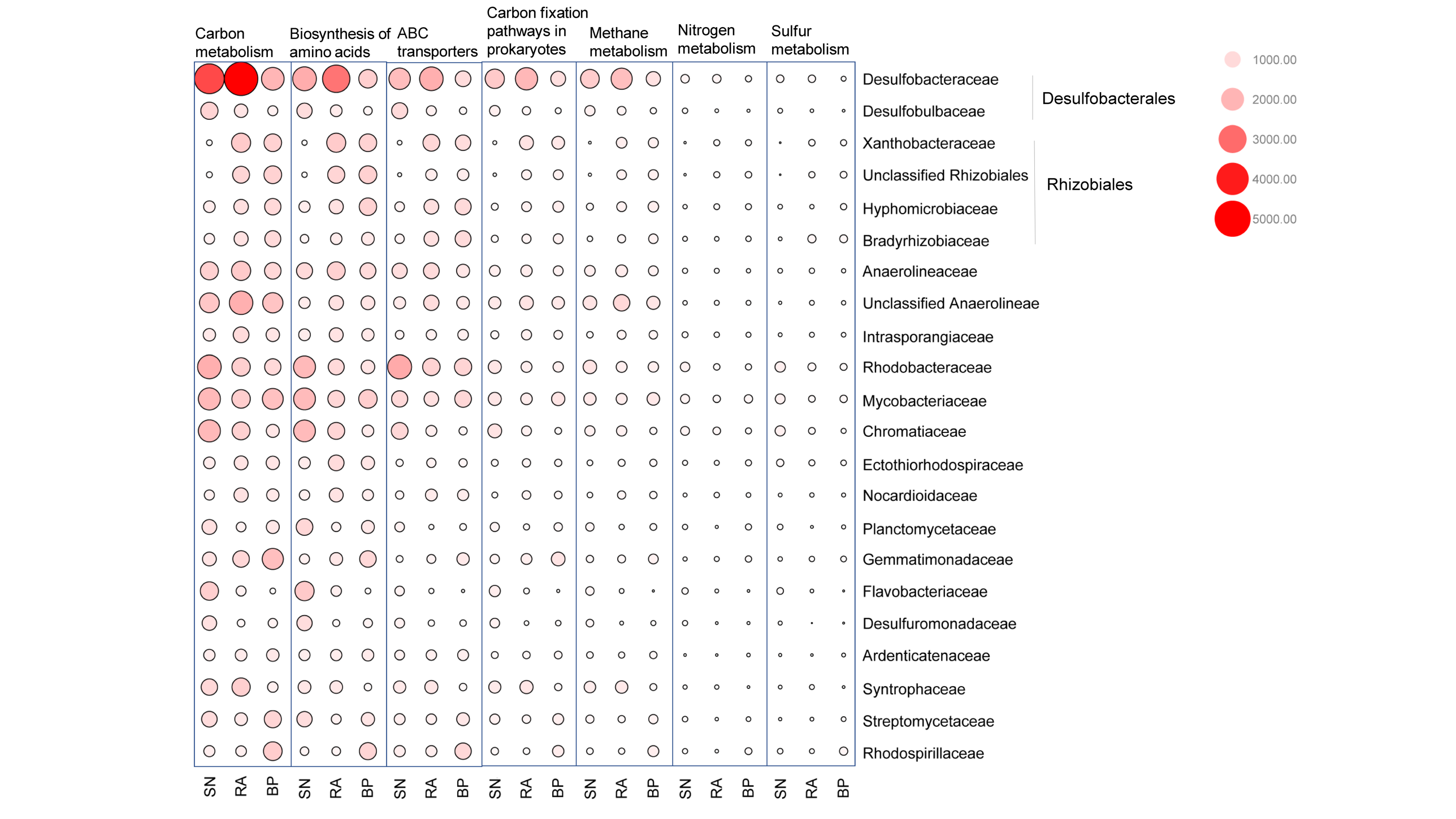

Supplement: Supplementary file 1 [file Data_Sheet_1.DOCX]
